# Fractal dimension shows reduced cortical complexity in Dementia with Lewy Bodies

**DOI:** 10.1002/alz70856_105988

**Published:** 2026-01-07

**Authors:** Kiran Aftab, Marcella Montagnese, Timothy Rittman

**Affiliations:** ^1^ University of Cambridge, Cambridge, Cambridgeshire, United Kingdom; ^2^ University of Cambridge, Cambridge, United Kingdom; ^3^ Department of Clinical Neurosciences, University of Cambridge, Cambridge, Cambridgeshire, United Kingdom; ^4^ Cambridge University Hospitals NHS Foundation Trust, Cambridge, United Kingdom

## Abstract

**Background:**

Fractal dimension (FD) is a measure of cortical complexity which is altered in ageing and other neurodegenerative disorders. It can serve as a useful structural marker to detect neurodegenerative changes in Dementia with Lewy bodies (DLB). We assessed the regional and whole brain fractal dimension indices in DLB in comparison to healthy controls and Alzheimer's disease (AD).

**Method:**

We included T1 weighted MRI scans of 87 DLB, 88 AD and 100 age‐ and gender‐matched healthy controls from the National Alzheimer's Coordinating Center (NACC), Neuroimaging of Inflammation in Memory and Related Other Disorders (NIMROD), Multimodal Imaging in Lewy Body Disorders (MILOS) and AMyloid imaging for Phenotyping LEwy body dementia (AMPLE) datasets. We segmented the cortical gray matter and white matter using Freesurfer version 7.3.4 and computed FD from the freesurfer outputs using the *fractalbrain* toolkit. Statistical analysis was performed using the R statistical package to explore group‐wise differences using MANOVA and Kruskal‐Wallis tests. The significance level was set at false‐discovery rate (FDR) corrected *p* < 0.05.

**Result:**

Cortical gray matter FD was significantly reduced globally as well as in bilateral frontal, temporal, parietal and occipital lobes in DLB compared to healthy controls. Moreover, FD was significantly lower in the cerebral white matter. Cortical gray matter FD was also reduced in AD compared to DLB globally and in bilateral parietal and left temporal lobes regionally. Cortical gray matter FD correlated with Addenbrooke's Cognitive Examination – Revised (ACE‐R) total scores and mean cortical thickness.

**Conclusion:**

Cortical complexity is reduced in the gray and white matter in DLB and can also be used to differentiate DLB from AD patients. Correlation with cognition and other structural markers may help in understanding disease‐specific structural changes.

